# A novel *Toxoplasma gondii* TGGT1_316290 mRNA-LNP vaccine elicits protective immune response against toxoplasmosis in mice

**DOI:** 10.3389/fmicb.2023.1145114

**Published:** 2023-03-21

**Authors:** Dan Li, Yizhuo Zhang, Shiyu Li, Bin Zheng

**Affiliations:** ^1^School of Basic Medical Sciences and Forensic Medicine, Hangzhou Medical College, Hangzhou, China; ^2^Engineering Research Center of Novel Vaccine of Zhejiang Province, Hangzhou Medical College, Hangzhou, China; ^3^Key Laboratory of Bio-tech Vaccine of Zhejiang Province, Hangzhou Medical College, Hangzhou, China

**Keywords:** *Toxoplasma gondii*, TGGT1_316290, mRNA vaccine, lipid nanoparticle, immune response

## Abstract

*Toxoplasma gondii* (*T. gondii*) can infect almost all warm-blooded animals and is a major threat to global public health. Currently, there is no effective drug or vaccine for *T. gondii*. In this study, bioinformatics analysis on B and T cell epitopes revealed that TGGT1_316290 (TG290) had superior effects compared with the surface antigen 1 (SAG1). TG290 mRNA-LNP was constructed through the Lipid Nanoparticle (LNP) technology and intramuscularly injected into the BALB/c mice, and its immunogenicity and efficacy were explored. Analysis of antibodies, cytokines (IFN-γ, IL-12, IL-4, and IL-10), lymphocytes proliferation, cytotoxic T lymphocyte activity, dendritic cell (DC) maturation, as well as CD4^+^ and CD8^+^ T lymphocytes revealed that TG290 mRNA-LNP induced humoral and cellular immune responses in vaccinated mice. Furthermore, T-Box 21 (T-bet), nuclear factor kappa B (NF-kB) p65, and interferon regulatory factor 8 (IRF8) subunit were over-expressed in the TG290 mRNA-LNP-immunized group. The survival time of mice injected with TG290 mRNA-LNP was significantly longer (18.7 ± 3 days) compared with the survival of mice of the control groups (*p* < 0.0001). In addition, adoptive immunization using 300 μl serum and lymphocytes (5*10^7^) of mice immunized with TG290 mRNA-LNP significantly prolonged the survival time of these mice. This study demonstrates that TG290 mRNA-LNP induces specific immune response against *T. gondii* and may be a potential toxoplasmosis vaccine candidate for this infection.

## Introduction

*Toxoplasma gondii* (*T. gondii*) is an obligate intracellular parasite that threatens one-third of the world’s population (Almeria et al., 2021; Smith et al., 2021). It has also become one of the most common zoonotic parasitic protozoa in the world because of its broad host range, high infection rate, and benign coexistence with the host (Montoya and Liesenfeld, 2004). Most people with normally functioning immune system show occult infection without clinical symptoms of *T. gondii* infection (Pittman and Knoll, 2015; Kochanowsky and Koshy, 2018). However, *T. gondii* seriously affects individuals with impaired immune systems, such as those with AIDS, malignancies, and organ transplant patients, and may cause death (Watts et al., 2015; Flegr and Escudero, 2016). Besides affecting newborns *in utero*, toxoplasmosis can also cause abortion in pregnant women (Robert-Gangneux and Dardé, 2012; Attias et al., 2020). In addition, *T. gondii* infection can be transmitted among livestock and pets, causing miscarriage and stillbirth, suggesting that it is major infection that causes severe economic losses in the livestock industry (Tenter et al., 2000). As a result, toxoplasmosis has attracted much attention in the medical and animal husbandry industry and has become a global public health concern (Montazeri et al., 2018).

Pyrimethamine and sulfadiazine are the current gold standard treatments for *T. gondii* infection (Alday and Doggett, 2017). However, increasing drug resistance has been detected in *T. gondii*, and thus could exacerbate the severity of the disease and lead to treatment failure (Wang et al., 2022). Although drug therapy is effective against *T. gondii* tachyzoites, it is ineffective against the cyst of *T. gondii* (Silva et al., 2021). This calls for development of effective vaccines for the long-term control and prevention of *T. gondii* infection while reducing the side effects and dependence on chemotherapy drugs (Wang et al., 2019).

To date, only one of commercially-licensed live attenuated vaccine were developed based on S48 strain (Toxovax®, MSD, New Zealand). Toxovax® is approved in a few regions (Europe and New Zealand) and can reduce the losses caused by congenital toxoplasmosis in sheep farming, indicating that *T. gondii* vaccine can be successfully exploited and commercially available for human immunization. Nevertheless, Toxovax® vaccine has some limitations, such as short-shelf life, uncertain genetic background, and reverting to the virulent wild type, making them unsuitable for further promotion and use.

Although several studies have employed various vaccination strategies, mainly including DNA vaccines, epitope vaccines, protein vaccines, inactivated vaccines, live vector-based vaccines, live attenuated vaccines, exosome vaccines, nanoparticle vaccines, and carbohydrate vaccines, to develop an effective toxoplasmosis vaccine (Wang et al., 2019), there is no safe and effective vaccine for *T. gondii* (Chu and Quan, 2021). mRNA vaccines are promising alternatives to conventional vaccines because of their high potency, low manufacturing cost, and short preparation period (Bok et al., 2021; Chaudhary et al., 2021). Nevertheless, only a few researches have analyzed mRNA vaccines against parasites.

This study aimed to explore a novel mRNA vaccine candidate against *T. gondii*. Furthermore, BALB/c mice were intramuscularly given TGGT1_316290 (TG290, ToxoDB accession number: TGGT1_316290) mRNA-LNP vaccine to evaluate its immunogenicity and immunoprotective effect.

## Materials and methods

### Epitope prediction

The potency of vaccine candidate antigens against *T. gondii* was assessed using PROTEAN program in DNASTAR software (Madison, Wisconsin, United States) based on its surface probability, flexible regions, antigenic index, and hydrophilicity. Additionally, the predicted half maximal inhibitory concentration (predicted IC50) values of polypeptides that bind to the major histocompatibility complex (MHC) class II molecules of vaccine candidate antigens were determined using Immune Epitope Database (IEDB, http://tools.immuneepitope.org/mhcii/).

### Mice, parasites, and cells

Specific-pathogen-free (SPF) male BALB/c mice (seven-week-old) were obtained from the Zhejiang Experimental Animal Center (Zhejiang province, China) and kept under standard routine conditions. Some mice were used in challenge test after immunization with mRNA vaccine, and others were used in challenge test after adoptive immunization. This study was approved by Hangzhou Medical College Institutional Animal Care and Use Committee (No: 2021-152) and followed the Chinese legislation regarding the use and care of research animals (GB/T35823-2018).

The *T. gondii* RH strain (type I) was stored at the laboratory for the extraction of total RNA, challenge experiments, and preparation of soluble tachyzoite antigens (STAg), as previously described (Zheng et al., 2017).

Vero, HEK293, and C2C12 cells preserved in the research group and maintained in DMEM containing 10% fetal bovine serum (FBS; Gibco, New Zealand) in an incubator with 5% CO_2_ at 37°C, were used for *T. gondii* generation and *in vitro* transfections.

### Expression of the recombinant TG290 (rTG290) protein and preparation of rabbit anti-TG290 polyclonal antibodies (rabbit anti-TG290 pAb)

The TG290 gene was amplified by PCR using *T. gondii* cDNA as template. TG290 and plasmid pET-28a were digested with BamHI and XhoI restriction enzymes. Then, it was separated by agarose gel electrophoresis and purified by AxyPrep™ DNA Gel Extraction Kit (Axygen, California, United States). T4 DNA ligase (Takara, Dalian, China) was used to ligate the digested DNA and pET-28a. The ligated products were transformed into TOP10 competent cells. Kanamycin was used to screen positive colonies. Finally, the positive plasmids were identified by sequencing. The constructed recombinant pET28a-TG290 vector was transformed into *E. coli* BL21 (DE3) to induce rTG290 expression overnight at 37°C *in vitro* using 0.1 mM/L isopropyl-beta-D-Thiogalactoside (IPTG). The bacteria were centrifuged at 12,000 × *g*, 4°C for 15 min and resuspended with PBS. Cells were cracked by ultrasound at low temperature (power: 200 W, 5 s ultrasonic interval 10s, 100 times), and supernatant was collected. Purified rTG290 was obtained using Ni2^+^-NTA agarose columns (Sangon Biotech, Shanghai, China) *via* affinity chromatography, then stored at −80°C for further use. The purified rTG290 was utilized to generate rabbit anti-TG290 polyclonal antibodies as described by Zheng et al. (2019).

### Formulation of TG290 mRNA and TG290 mRNA-LNP

Wild-type constructs encoding TG290 [containing a T7 promoter site for *in vitro* transcription (IVT) of mRNA, 5′ and 3′ UTRs] were synthesized by GenScript Biotech Corporation (Nanjing, China). The −300 bp upstream and 300 bp downstream sequences of the TG290 gene were used for the 5′ UTR and 3′ UTR, respectively. The T7 RNA polymerase promoter site upstream of the 5′ UTR is used to transcribe mRNA *in vitro*. Standard mRNA was synthesized from linearized DNA using the T7 *in vitro* transcription kit with unmodified nucleotides (CELLSCRIPT™, Wisconsin, United States). Incognito mRNA Synthesis Kit (CELLSCRIPT™, Wisconsin, United States) was used to generate RNA encapsulated in lipid nanoparticles with pseudouridine instead of uridine. NanoAssemblr® Benchtop system (Precision Nanosystems Inc., PNI, British Columbia, Canada) was used to encapsulate enzymatically added structures of 5′ cap-1 and 3′ poly (A). The mRNA was solubilized with PNI formulation buffer (PNI, British Columbia, Canada). mRNA-LNPs were generated at a 3:1 flow rate ratio (RNA in PNI buffer; GenVoy-ILM™) through a laminar flow cassette at 12 ml/min. Quant-iT™ RiboGreen™ RNA Reagent and Kit (Invitrogen, California, United States) was used to assess the encapsulation efficiency and mRNA concentration of mRNA-LNPs.

### *In vitro* transfections

HEK293 and C2C12 cells were transfected with TG290 mRNA using Lipo2000 reagent (Invitrogen, California, United States) following the manufacturer’s protocol. Cells were seeded in 12-well plates until they reached 70–90% confluence after transfection. They were transferred to a fresh DMEM medium. 4 μl Lipo 2000 liposomes were added to 100 μl Gibco Opti MEM medium and mixed well for 5 min at room temperature. 2 μg mRNA was added to 100 μl Opti MEM medium and mix well for 5 min at room temperature. The mRNA-lipid complexes were mixed together at room temperature for 20 min and then carefully added to the culture medium. The cell supernatant and whole cell lysate were collected 24 h after transfection for subsequent experiments. The cells were washed with phosphate-buffered saline (PBS) and lysed with radioimmunoprecipitation (RIPA) buffer (Biotime, Haimen, China), then centrifuged at 16,000 × *g*, 4°C for 10 min. The supernatant was purified *via* ultracentrifugation at 140,000 × *g* overnight (16 h) at 4°C using a 20% sucrose cushion. Protein complexes were resuspended in 50 μl of PBS containing 1% bovine serum albumin (BSA) and stored at −20°C for subsequent analysis. Lysates or purified supernatant samples (10 μl) were detected *via* western blotting.

### Immunization and *Toxoplasma gondii* challenge

Seven-week-old male BALB/c mice (*n* = 60) were randomly divided into three groups (experimental group, negative group, and blank group) to evaluate the prophylactic efficacy of TG290 mRNA-LNP. The mice in the experimental group (*n* = 20) were intramuscularly given 10 μg TG290 mRNA-LNP vaccine once every 2 weeks (thrice), while the mice in the negative group (*n* = 20) were injected with empty LNP. The mice in the blank group were not given any treatment (*n* = 20). Blood was collected from each mouse on days 0, 13, 27, and 41, and the serum was stored at – 20°C. The mice were intraperitoneally challenged with 100 tachyzoites of highly virulent *T. gondii* RH strain at 2 weeks after final administration (15 mice per group), then survival time was monitored daily.

Furthermore, serum was collected from each group (*n* = 5) in the immunization section to assess the protective effect of serum transfer immunization. The naive mice (*n* = 30) were divided into three groups, then injected with TG290 mRNA-LNP-immunized serum (*n* = 10), LNP-immunized serum (negative group, *n* = 10), and the naive serum (blank group, *n* = 10) *via* (100 μl of serum per mice) tail vein daily for 5 days. The mice were also intraperitoneally injected with 100 RH tachyzoites and the survival time was monitored.

Splenocytes were obtained from each group of mice (*n* = 5) in the immunization section to evaluate the preventive effect of splenocyte transfer immunization. The naive mice (*n* = 30) were divided into three groups. The mice were injected with TG290 mRNA-LNP-immunized splenocytes or LNP-immunized splenocytes, or naive splenocytes *via* the tail vein (5*10^8^ splenocytes per mouse). The mice were also given *T. gondii* RH strain (100 tachyzoites) intraperitoneally at 24 h post injection, and the survival time was monitored daily.

### Indirect ELISA

Each hole in the ELISA board was coated with rTG290 (20 μg/ml) and incubated overnight in a 2–8°C wet box. The plates were washed five times with PBST (0.05% Tween-20 in PBS) and blocked with 1% bovine serum albumin (BSA) at room temperature for 2 h. 100 μl diluted serum (1:100) was added to each well and incubated at room temperature for 2 h. The plates were washed five times with PBST (0.05% Tween-20), then the samples were incubated with 100 μl 1:100000 diluted horseradish peroxidase (HRP) labeled Goat Anti-Mouse IgG, IgG1, or IgG2a (Abcam, Cambridge, United Kingdom) at 37°C for 1 h in each well. The plates were washed again, and the samples were incubated with 100 μl tetramethylbenzidine substrate reaction solution at room temperature and away from light for 15 min in each well. An automatic ELISA reader (BioTek, Virginia, United States) was used to determine the OD value of the sample at 450 nm. Meanwhile, the corresponding blank control group and negative control group were set up. All samples were in triplicates.

### Preparation of spleen cell suspension

Spleen was removed from five mice in each group after 2 weeks of the last vaccination, then ground with nylon sieve to obtain splenocytes. Red blood cell lysis buffer (Sigma, Melbourne, United States) was added to remove blood cells. The spleen cells were resuspended in Dulbecco’s modified Eagle’s medium with 10% FBS (Gibco, New Zealand). The suspension of 45 μl splenic lymphocytes was mixed with 5 μl 0.4% trypan blue dye and stained under a microscope (count of viable cells should be above 95% before the next experiment).

### Lymphocyte proliferation assay

Cell Counting Kit-8 (CCK-8, Dojindo, Kumamoto, Japan) was used to determine the proliferation activity of spleen lymphocytes following the manufacturer’s instructions. About 2*10^5^ purified spleen lymphocytes per well were incubated into 96-well microplates. The lymphocytes were stimulated with rTG290 (10 μg/ml). Concanavalin A (ConA; 5 μg/ml; Sigma) was added as a positive control, and Dulbecco’s modified Eagle’s medium alone was added as a negative control. CCK-8 (Dojindo, Kumamoto, Japan) was added to each well after 4 days, then incubated at 37°C for 4 h for lymphocyte proliferation. The stimulation index was calculated as follows: stimulation index (SI) = (OD_570_ rTG290 − OD_570_ Control): (OD_570_ ConA − OD_570_ Control).

### Cytokine measurement

Splenic lymphocytes (100 μl: adjusted density of 5*10^6^ cells/mL) were added to each well with 96-well plates, followed by ConA (5 μg/ml) or rTG290 (10 μg/ml) addition. The cells were then cultured in a 5% CO_2_ incubator at 37°C. Each BABL/c mouse in each group had three parallel wells. The cell supernatants were used to evaluate the level of IL-4 at 24 h, IL-10 at 72 h, and IFN-γ and IL-12 at 96 h. Mouse ELISA Kit with Pre-coated Plates series (eBioscience, California, United States) was used to for cytokine measurement following the manufacturer’s instructions.

### Cytotoxic T lymphocyte activity assays

Cytotoxic T-lymphocyte (CTL) assay was performed *via* lactate dehydrogenase-release assay using CytoTox 96® Non-Radioactive Cytotoxicity Assay Kit (Promega, Wisconsin, United States). Lymphocytes were cultured with 100 U/ml recombinant mouse IL-12 (eBioscience, California, United States) for 5 days to act as effector cells, while Sp2/0 cells were transfected with TG290 mRNA-LNP to serve as target cells. Effector cells and target cells were mixed at a scale of 10:1, 20:1, 40:1, 80:1, and incubated at 37°C for 6 h. Cytotoxicity was calculated as follows: %Cytotoxicity = (Experimental -Effector Spontaneous-Target Spontaneous)/(Target Maximum-Target Spontaneous) × 100.

### Flow cytometry assay

The percentages of CD4^+^ and CD8^+^ T cells of mice in the TG290 mRNA-LNP, LNP, and blank groups were detected using flow cytometry. Splenic lymphocytes were harvested and dead cells were first removed using the Dead Cell Removal Kit (Miltenyi Biotec, no. 130-090-101) following the manufacturer’s instructions. Surviving cells were counted and single cell suspensions were prepared by resuspending them at 1 × 10^7^ cells/ml in cell staining buffer. They were then pre-incubated with TruStain FcX™ PLUS (anti-mouse CD16/32) antibody at 2.5 μg per 10^7^ cells in a volume of 100 μl for 5–10 min on ice to block the Fc receptor. Subsequently, the cells were incubated with anti-mouse CD3e-FITC + anti-mouse CD8-PE and anti-mouse CD3e-FITC + anti-mouse CD4-PE antibodies (Abcam, Cambridge, United Kingdom) in the dark at 4°C for 30 min. For CD83, CD86, and MHC molecule changes in splenic DCs, the separated lymphocytes were double-stained with CD11c-FITC + CD83-PE, CD11c-FITC + CD86-PE, CD11c-FITC + MHC-I-PE, and CD11c-FITC + MHC-II-PE (eBioscience, California, United States) in the dark at 4°C for 30 min. Cell populations were analyzed *via* flow cytometry using FACScan flow cytometer (Becton Dickinson, New Jersey, United States) and CellQuest software (Becton Dickinson, New Jersey, United States).

### Cytokine-related transcription factor assay

qRT-PCR and western blotting were used to assess the expression levels of interferon regulatory factor 8 (IRF8), T-Box 21 (T-bet), and nuclear factor kappa B (NF-kB) p65 in prepared splenocyte lymphocytes. Total RNA was isolated from the cells using a RNAsimple Total RNA kit (TaKaRa, Dalian, China). It was then used to synthesize cDNA with the ReverTra Ace -a-™ (Toyobo, Osaka, Japan) kit whereas the KOD SYBR® qPCR Mix (Toyobo, Osaka, Japan) was used to quantify mRNA expression following the manufacturer’s protocol. The qPCR program was as follows: pre-denaturation at 98°C for 2 min, denaturation at 98°C for 10 s, annealing at 60°C for 10 s, and extension at 68°C for 30 s (40 cycles). The primers utilized are shown in Table 1. Western blotting was used to analyze transcription factors (IRF8, T-bet, NF-kB p65) in the nucleus *via* the Nuclear and cytoplasmic Isolation Kit (Biotime, Haimen, China). The antibodies used in this study were source from Cell Signaling Technology, Inc. (Danvers, MA, United States) with the following catalog numbers: #5628 for IRF8, #13232 for T-bet, #8242 for NF-kB p65, and #4499 for H3 Histone.

**Table 1:** RT -PCR primers used to amplify the NF-κB p65, T-bet, IRF8, and β-actin genes designed by primer premier 6.0.

Primer name	Sequence
NF-κB p65-F	5′-GAACCAGGGTGTGTCCATGT-3′
NF-κB p65-R	5′-TCCGCAATGGAGGAGAAGTC-3′
T -bet-F	5′-GCCAGGGAACCGCTTATATG-3′
T -bet-R	5′-TGGAGAGACTGCAGGACGAT-3′
IRF8-F	5′-GCTGATCAAGGAACCTTGTG-3′
IRF8-R	5′-CAGGCCTGCACTGGGCTG-3′
β-Actin-F	5′-GCTTCTAGGCGGACTGTTAC-3′
β-Actin-R	5′-CCATGCCAATGTTGTCTCTT-3′

### Statistical analysis

GraphPad Prism 8.0 (GraphPad, California, United States) was used for statistical analyses. One-way ANOVA with multiple comparisons (compare the mean of each group with the mean of every other group) was used to assess the differences in antibody levels, cytokine levels, lymphocyte proliferation, and flow cytometry assays among all the groups. Log-rank tests were used to evaluate the survival time. *p* < 0.05 was considered statistically significant.

## Results

### Bright B cell and T cell epitopes prediction of TG290

**Figure 1:**
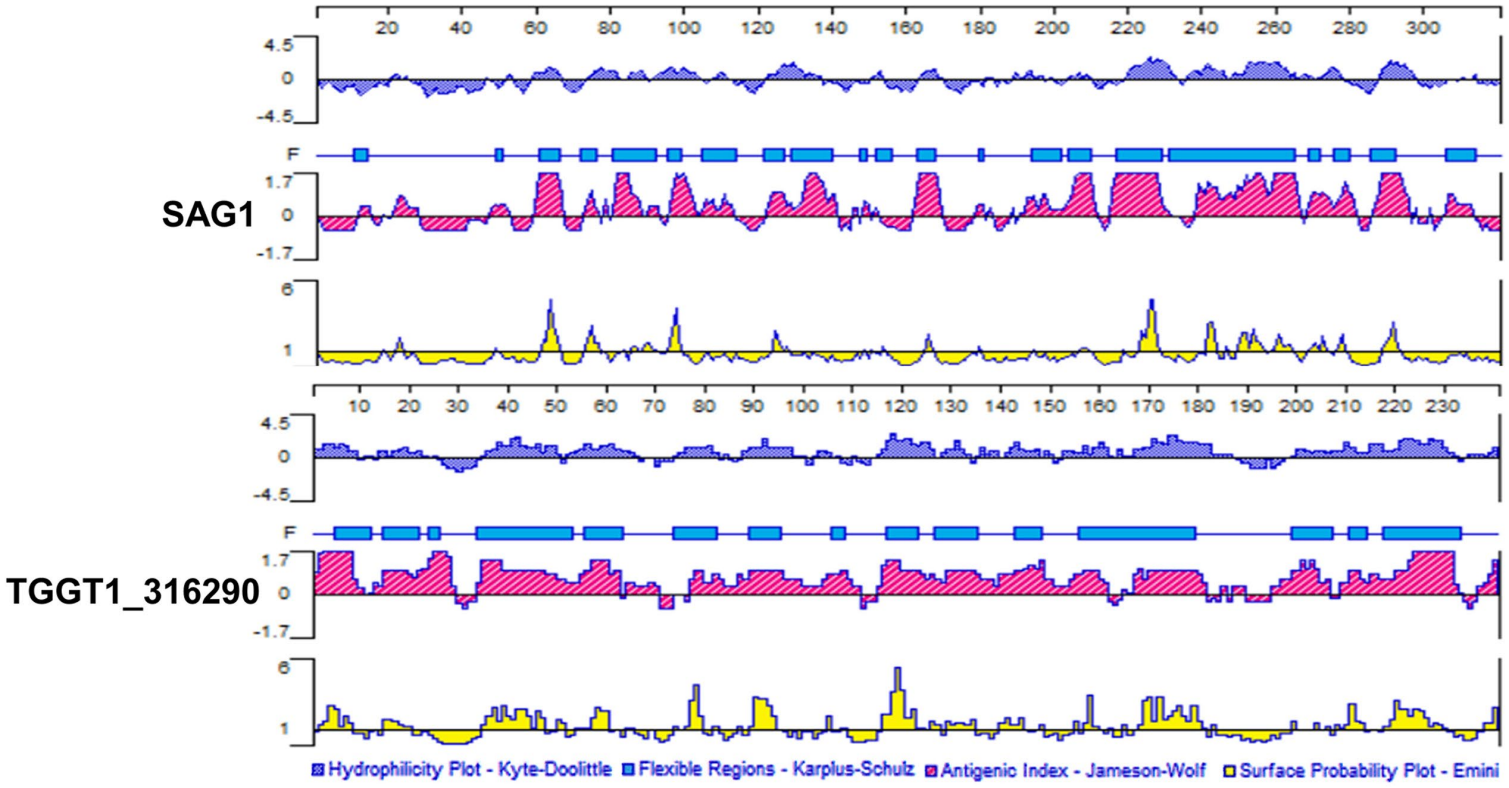
Comparison of linear-B cell epitopes of TG290 and SAG1 in terms of DNASTAR predictions of hydrophilicity, flexible regions, antigenic index, and surface probability.

Antigenic epitopes generally have high hydrophilicity, flexibility, antigen index and multi-surface probability (Lyn et al., 1991). Hydrophilic amino acids are enriched on the surface of the hydrophilic region, and these sites have evolutionary been used as the main amino acid insertion sites for protein, which also lays a foundation for the secretion of the protein into the cytoplasm and extracellular. The amino acid residues with strong flexibility are the sites with high plasticity, which can easily form antigenic epitopes. Surface probability illustrates the probability that the antigenic point is located in the exposed area of the protein surface. The antigen index can reflect the antigenicity scale by analyzing the amino acids of the continuous sites of the well-studied proteins, and the value can be derived by dividing the frequency of each amino acid in all proteins by the frequency at which each amino acid appears in the antigen region. PROTEAN program in DNASTAR software was used to analyze B cell epitopes of TG290 and SAG1. The regions with hydrophilicity (the critical value is 0) of TG290 are 1–10, 15–16, 17–23, 36–51, 52–67, 75–83, 88–98, 103–106, 115–127, 128–134, 136–140, 141–148, 149–151, 153–162, 164–165, 166–187, 200–232, 235–240. The surface probability regions (the critical value is 1) of TG290 are 3–9, 15–20, 35–47, 50–51, 54–60, 76–79, 89–94, 105, 116–122, 126–127, 129–134, 140–144, 149, 157–159, 168–181, 208–213, 218–230, 238–240. The antigen index regions of TG290 (the critical value is 0) are 1–10, 13–29, 34–62, 64–70, 77–111, 115–161, 167–181, 187–189, 195–206, 209–232, 237–240. The flexibility regions of TG290 are 5–12, 15–22, 24–26, 34–53, 56–63, 74–82, 89–95, 106–108, 117–123, 127–135, 143–148, 156–179, 199–207, 211–214, 218–233. TG290 linear-B cell epitopes were more excellent than SAG1 in terms of surface probability, antigenic index, flexibility, and hydrophilicity (Figure 1). In addition, IEDB online service was used to evaluate T cell epitopes of TG290 and SAG1 as the published article (Fleri et al., 2017). Although the predicted half-maximal inhibitory concentration (predicted IC50) of H2-IAb of TG290 was higher than that of SAG1, the predicted IC50 of H2-Iad and H2-Ied of TG290 was significantly lower than that of SAG1. The predicted IC50 values of HLA-DRB 1*01:01 were similar for both TG290 and SAG1. The results revealed that TG290 protein may have strong binding to MHCII (Table 2). Bioinformatic analysis showed that TG290 peptides had an ideal score of linear-B cell epitopes and a lower percentile of predicted IC50 values than SAG1, suggesting that TG290 has a promising prospect for generating vaccine.

**Table 2:** Predicted IC50 values from IEDB^a^ for TG290 and SAG1 binding to MHC class II molecules.

MHC II alblele^b^	Start-stop^c^		Percentile rank^d^	
	SAG1	TG290	SAG1	TG290
H2-IAb	26–40	28–42	0.95	3.6
H2-IAd	170–184	48–62	2.45	0.49
H2-IEd	14–28	18–32	3.35	0.51
HLA-DRB 1*01:01	12–26	16–30	1.8	2.1

a The immune epitope database (http://tools.immuneepitope.org/mhcii).

b H2-IAb, H2-IAd, and H2-IEd alleles are mouse MHC class II molecules; the HLA-DRB1*01:01 allele is a human MHC class II molecule.

c 15 amino acids were chosen for analysis.

d Low percentile indicates high level binding according to the software instructions.

In addition, we used BepiPred-2.0 and NetChop for further analysis of TG290 and SAG1 epitopes. The phylogenetic tree of TG290 was constructed using MEGA software. SWISS – MODEL^1^ to build TG290 protein structure. The structural models of mouse BCR(No.8EMA), TCR (No.8D5P) and MHCII-TCR complex (No.3C60) were obtained from the RCSB PDB.^2^ Cluspro 2.0^3^ protein–protein Docking was used to construct the spatial interaction between TG290 and BCR, TCR and MHCII-TCR. We have provided these results in the Supplementary materials.

### Preparation and delivery of TG290 mRNA-LNP

**Figure 2:**
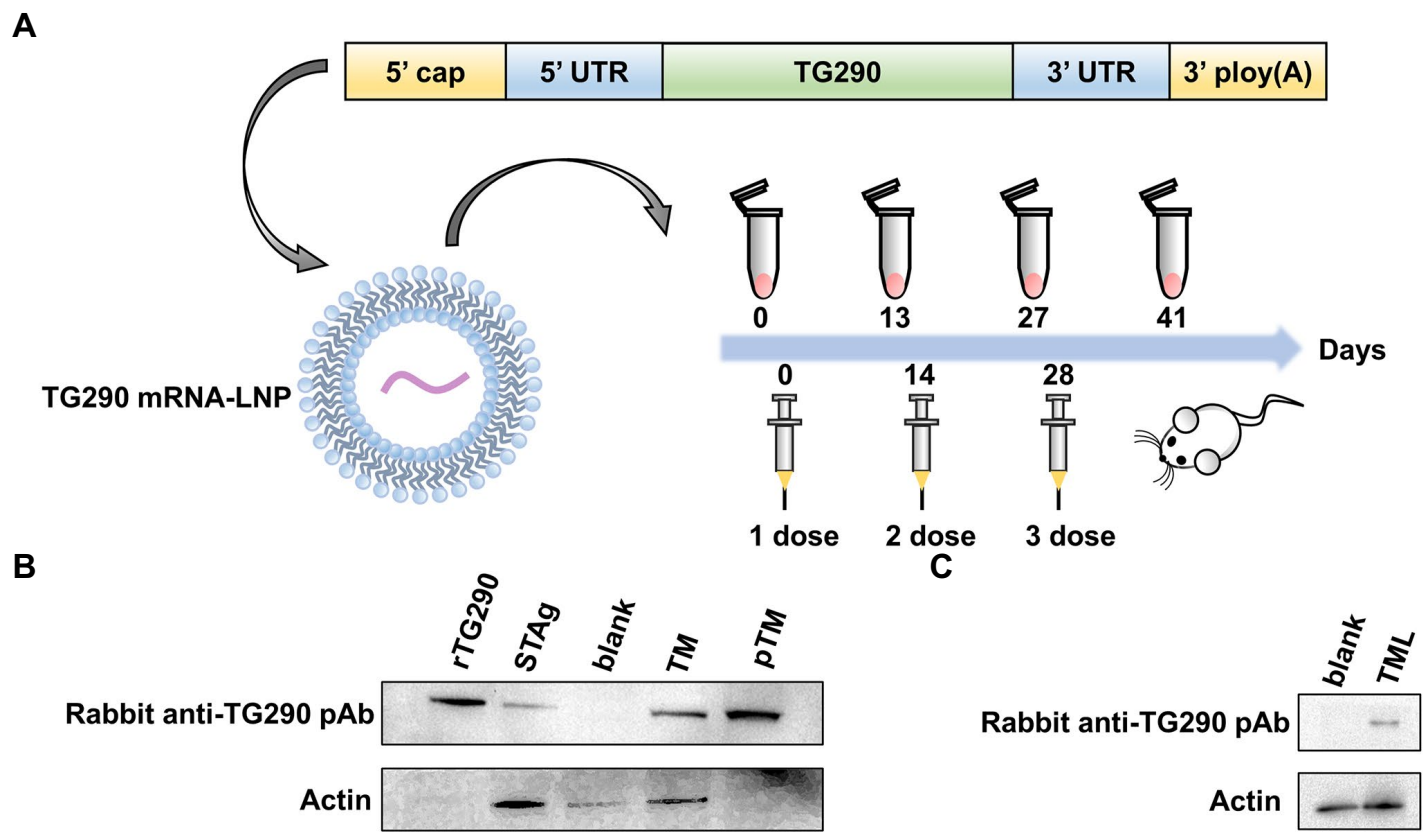
The construction, protein expression and delivery of TG290 mRNA-LNP vaccine. **(A)** The engineered mRNA construct. An mRNA encoding the TG290 was designed with 5′ and 3′ untranslated regions (UTRs) flanking the coding sequence, a 5′ cap-1 structure and a 3′ poly(A) tail. *In vitro*-synthesized mRNA encapsulated in a lipid nanoparticle to be applied in *in vitro* and *in vivo* experiments. **(B)** The expression of TG290 in HEK293 cell lysates and STAg was determined by Western blot. rTG290 served as a positive control; STAg: (soluble antigen of *T. gondii*); Blank: non-transfected cells; Unpurified cell lysate from TG290 mRNA-transfected cells ©; Purified supernatant from TG290 mRNA-transfected cells (pTM). **(C)** *In vitro*-synthesized TG290 mRNA was encapsulated in a lipid nanoparticle and administered to C2C12 cells. Lysate was analyzed by Western blotting with Rabbit anti-TG290 pAb. Blank: empty transfected cells; TG290 mRNA-LNP transfected cells (TML).

Mature mRNAs similar to host mRNAs were generated. The essential structure of TG290 mRNA consists of the 5′, 3′ UTRs, 5′ cap-1 structure, and a 3′ poly (A) tail (Figure 2A).

The rabbit anti-TG290 pAb can pick out rTG290 (Figure 2B). Meanwhile, the native TG290 was explicitly recognized in STAg. *In vitro*-synthesized mRNA was transfected into HEK293 cells, and the cell lysate and supernatant were collected to further identify mRNA expression. The purified supernatants did not contain β-actin band, demonstrating that any cytoplasmic contamination was removed *via* ultracentrifugation (Figure 2B). In addition, a single specific TG290 band was detected in lysate and supernatant of cells transfected with TG290 mRNA, while no band was detected in the blank control group (Figure 2B). Taken together, these findings show that mRNAs synthesized *in vitro* can induce TG290 protein expression.

Invitrogen’s Quant-iTRibogreen RNA assay kit (Invitrogen, California, United States) detected encapsulation efficiency of 95.8%. Furthermore, protein expression in muscle cells was characterized after administration of TG290 mRNA-LNP *via* intramuscular injection to differentiated skeletal myoblasts C2C12 cells, which induced TG290 expression (Figure 2C).

### TG290 mRNA-LNP facilitates the production of TG290-specific total IgG and subtype IgG1, IgG2a antibodies

**Figure 3:**
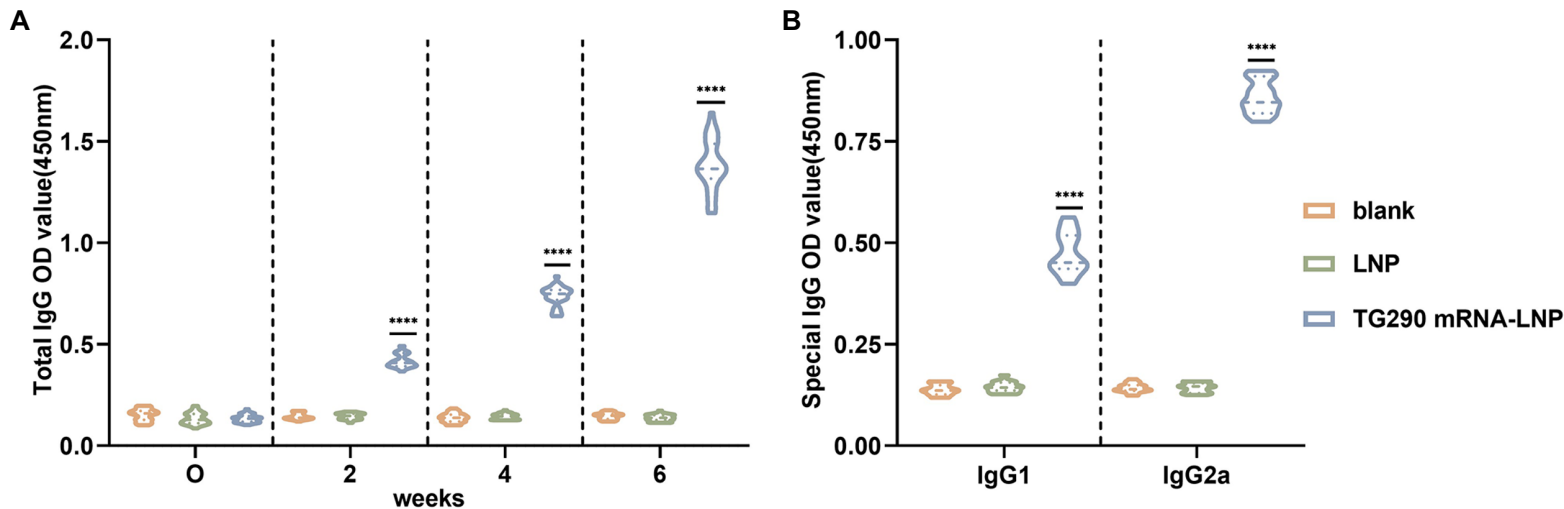
TG290 mRNA-LNP vaccine elicited humoral immunity response in BALB/c mice. **(A)** Total TG290-specific IgG; **(B)** TG290-specific IgG1, IgG2a. The OD450 value of total TG290-specific IgG was recorded at 0-, 2-, 4-, and 6-weeks post-vaccination (*n* = 10). The OD450 value of TG290-specific IgG subclass (IgG1, IgG2a) was recorded at 6 weeks post-vaccination (*n* = 5). ****p < 0.0001, analyzed by One-way ANOVA with multiple comparisons (compare the mean of each group with the mean of every other group).

The levels of TG290-specific total IgG, IgG1, and IgG2a were determined *via* enzyme-linked immunosorbent (ELISA) assays to evaluate whether TG290 mRNA-LNP can elicit specific humoral immune response. The TG290-specific total IgG antibody titer increased with the number of injections (Figures 2A, 3A). Furthermore, serum antibody titers were substantially higher in mice vaccinated with TG290 mRNA-LNP than in the control groups (*p* < 0.0001, Figure 3A). The levels of TG290-specific IgG1 and IgG2a were considerably increased in mice immunized with TG290 mRNA-LNP vaccine compared with the blank and LNP groups (*p* < 0.0001, Figure 3B). Notably, the IgG2a levels (0.86 ± 0.01, *p* < 0.0001, Figure 3B) were significantly higher than IgG1 level (0.47 ± 0.01, *p* < 0.0001, Figure 3B).

### Generation of high levels cytokines (IFN-γ, IL-12, IL-4, and IL-10) in mice vaccinated with TG290 mRNA-LNP

**Figure 4:**
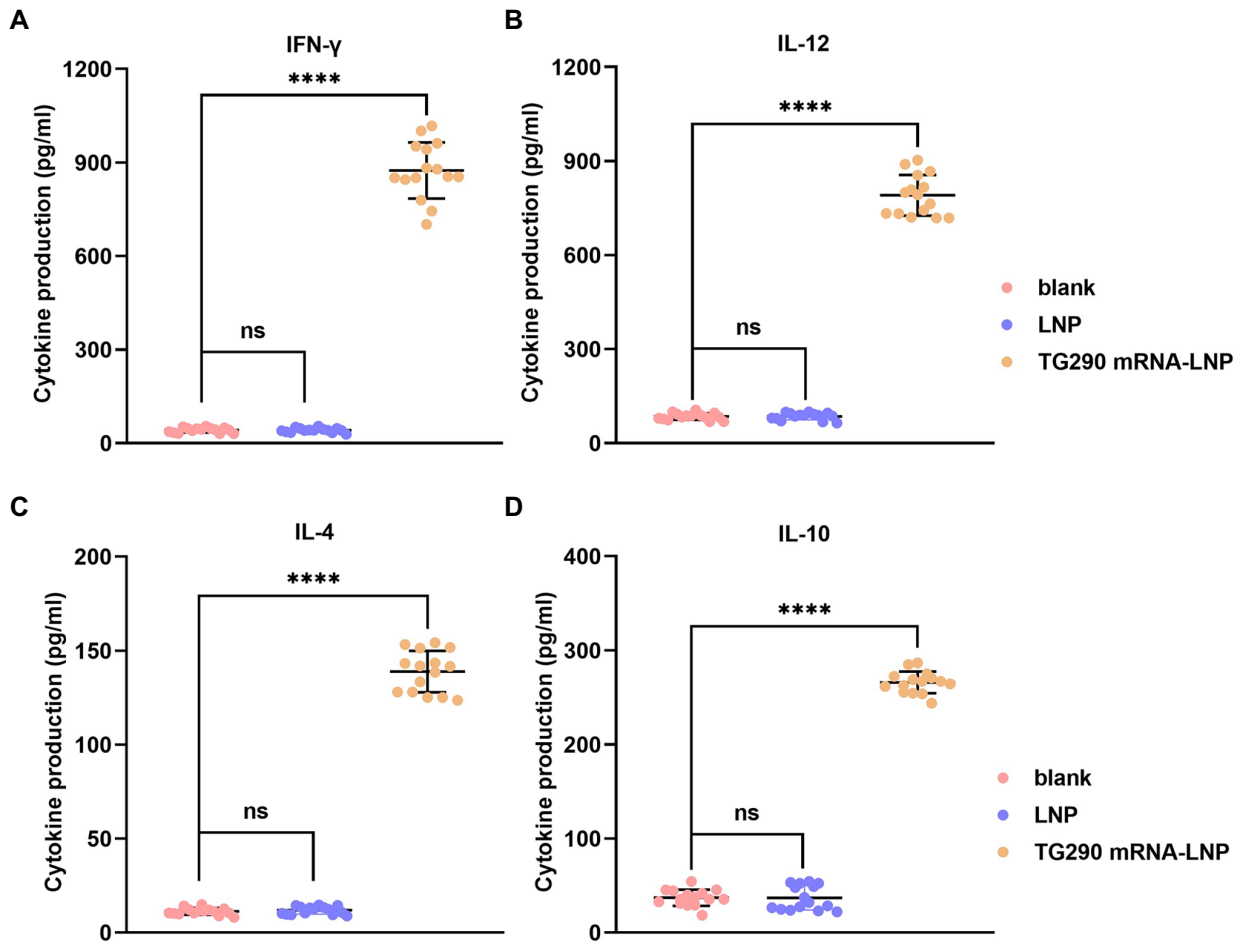
The determination of cytokines. The prepared spleen cell suspension was obtained from mice 2 weeks of the last vaccination. The levels of **(A)** IFN-γ and **(B)** IL-12 and **(C)** IL-4 and **(D)** IL-10 in BALB/c mice immunized with TG290 mRNA-LNP vaccine. Data were obtained from triplicate independent experiments and are presented as means ± SD (*n* = 5). ****p < 0.0001, analyzed by one-way ANOVA multiple comparisons (compare the mean of each group with the mean of every other group).

The levels of Cytokines (IFN-γ, IL-12, IL-4, and IL-10) were measured to determine the type of T helper cell response. The levels of IFN-γ (874.6 ± 27.1 pg./ml), IL-12 (790.6 ± 9.8 pg./ml), IL-4 (138.8 ± 1.7 pg./ml), and IL-10 (265.9 ± 2.3 pg./ml) were significantly elevated in mice immunized with TG290 mRNA-LNP vaccine compared with the control groups (*p* < 0.0001; Figure 4). Collectively, these results reveal that TG290 mRNA-LNP elicits a mixed Th1/Th2 response.

### TG290 mRNA-LNP vaccination enhances lymphocyte proliferation ability and CTL activity

**Figure 5:**
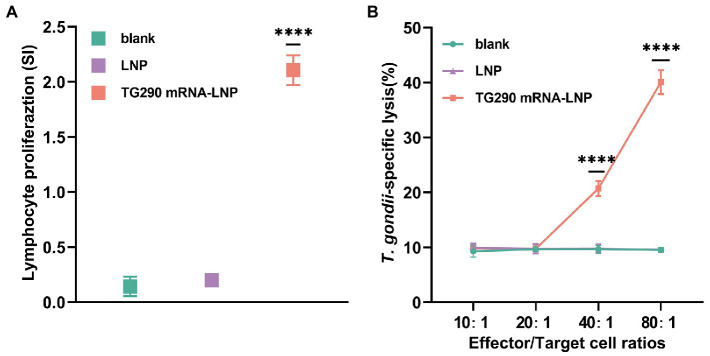
Proliferation and CTL activity of lymphocytes in immunized BALB/c mice. **(A)** The proliferative responses of splenocytes in immunized BALB/c mice. SI, stimulation index. **(B)** CTL activity of spleen lymphocytes in TG290 mRNA-LNP immunized mice. The horizontal axis indicates the effector-to-target cell ratios. The vertical axis shows the percentage of *T. gondii*-specific lysis. Data were obtained from triplicate independent experiments and are presented as means ± SD (*n* = 5). ****p < 0.0001, analyzed by One-way ANOVA with multiple comparisons (compare the mean of each group with the mean of every other group).

Splenocytes were obtained at 2 weeks post-final immunization to assess whether the splenic T lymphocytes were validly activated. Lymphocyte proliferation was measured with rTG290 as a stimulator, medium only as a negative control, and ConA as positive control. The stimulation index (SI) of lymphocytes was considerably higher in BALB/c mice vaccinated with TG290 mRNA-LNP (SI: 2.1 ± 0.06, Figure 5A, *p* < 0.0001) than mice in the blank and LNP groups.

CTL responses are crucial for efficient protection against *T. gondii*. Herein, CTL activity of splenic lymphocytes was higher in mice vaccinated with TG290 mRNA-LNP than in the control group when effector target ratio was 40:1 and CTL activity was highest at 80:1 (Figure 5B, *p* < 0.0001).

### Augmentation in splenic T lymphocytes and dendritic cells levels post TG290 mRNA-LNP vaccine immunization

**Figure 6:**
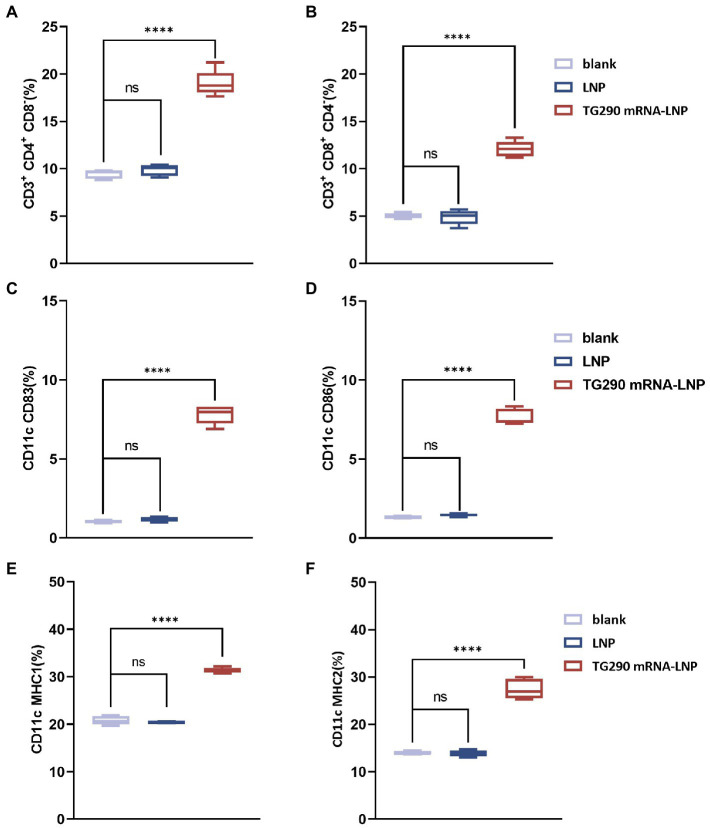
Flow cytometry analysis. The percentage of CD4+ **(A)** and CD8+ T lymphocytes **(B)** in splenic lymphocytes. The percentage of CD83 **(C)** and CD86 molecules **(D)** on splenic DCs. The percentage of MHC-I **(E)** and MHC-II molecules **(F)** on splenic DCs. *n* = 5; ****p < 0.0001, analyzed by One-way ANOVA with multiple comparisons (compare the mean of each group with the mean of every other group).

The splenic lymphocytes were harvested at 2 weeks after the last immunization to investigate the effect of TG290 mRNA-LNP on T Lymphocytes. The levels of CD4^+^CD8^−^ (Figure 6A) and CD8^+^CD4^−^ (Figure 6B) T lymphocytes were higher in mice vaccinated with TG290 mRNA-LNP than in the blank and LNP groups (*p* < 0.0001).

Meanwhile, CD83 (Figure 6C) and CD86 (Figure 6D) levels on the surfaces of DCs were higher in mice inoculated with TG290 mRNA-LNP than in the blank and LNP groups (*p* < 0.0001). Additionally, TG290 mRNA-LNP vaccination significantly promoted increased levels of MHC-I (Figure 6E) and MHC-II (Figure 6F) molecules (*p* < 0.0001). Taken together, these results indicate that TG290 mRNA-LNP elicits higher levels of the CD83 and CD86 molecules of DCs and plays a critical role in the antigen presentation effects of DCs.

### TG290 mRNA-LNP immunization increases expression of cytokine-related transcription factors

**Figure 7:**
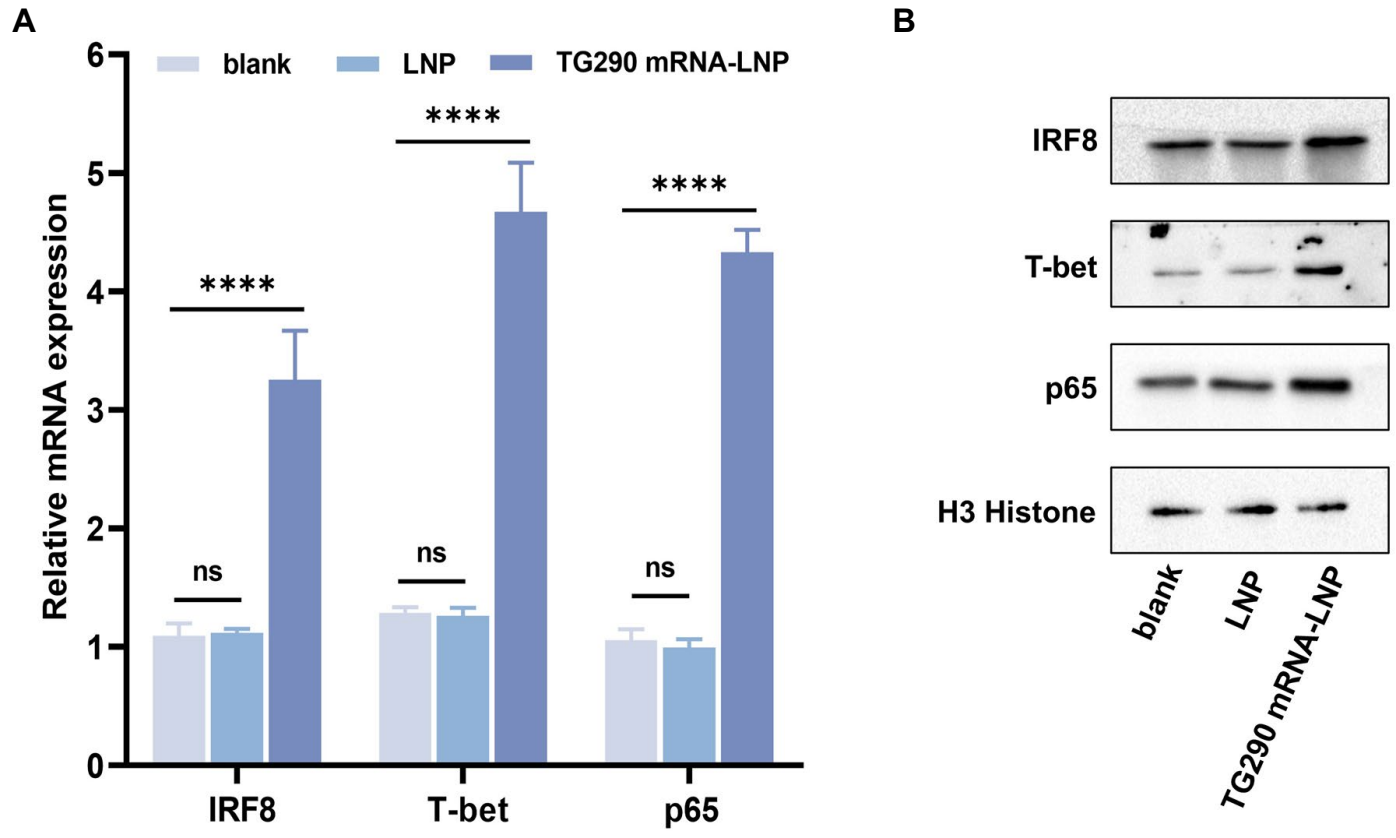
The mRNA and protein expression levels of IRF8, T-bet, and p65 in splenocytes. **(A)** The mRNA expression levels of IRF8, T-bet, and p65. **(B)** The protein expression levels of IRF8, T-bet, and p65. Data were obtained from triplicate independent experiments and are presented as means ± SD (*n* = 5). ****p < 0.0001, analyzed by One-way ANOVA with multiple comparisons (compare the mean of each group with the mean of every other group).

The mRNA and protein expression levels of IRF8, T-bet, and p65 were determined by qPCR and western blot, respectively. The expression levels of T-bet, p65, and IRF8 were markedly higher in mice vaccinated with TG290 mRNA-LNP than in the blank and LNP groups (Figure 7, *p* < 0.0001). These results demonstrated that TG290 mRNA-LNP can stimulate the expression of T-bet, p65, and IRF8.

### TG290 mRNA-LNP vaccination prolongs the survival time

**Figure 8:**
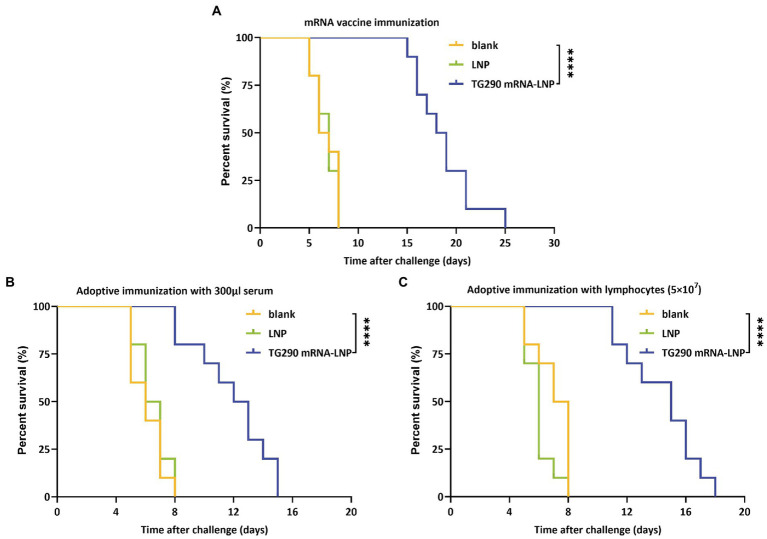
Survival curves of BALB/c mice after challenge. **(A)** TG290 mRNA-LNP vaccine protected against the lethal challenge (15 mice per group). **(B)** Adoptive immunization with serum protected against the lethal challenge (10 mice per group). **(C)** Adoptive immunization with lymphocytes protected against the lethal challenge (10 mice per group). ****p < 0.0001 by the log rank tests.

The survival curve (Figure 8) showed that TG290 mRNA-LNP-vaccinated mice (Figure 8A, 18.5 ± 3 days, *p* < 0.0001) had prolonged survival time than mice in the blank and LNP groups (The mice in the two groups died within 8 days). Meanwhile, the survival time of mice adoptively transferred using serum (Figure 8B, 11.9 ± 2.6 days, *p* < 0.0001) and splenocytes (Figure 8C, 14.4 ± 2.5 days, *p* < 0.0001) was longer than that of mice in the control group (died within 8 days). These datas demonstrated that TG290 mRNA-LNP vaccine prolonged the survival time of mice.

## Discussion

*T. gondii* is a major opportunistic pathogen infecting nearly more than two billion people worldwide (Hakimi et al., 2017). Vaccination can effectively prevent zoonoses (Rahman et al., 2020), especially mRNA vaccines since they have low insertional mutagenesis risk, safe delivery, low manufacturing cost, accelerated development cycles, and high potency. Various mRNA vaccines are at the clinical trial stage and can combat the emergence and re-emergence of infectious diseases, including rabies, Zika, and influenza (Le et al., 2022). Furthermore, mRNA vaccines have recently been successfully used against COVID-19, validating the platform and showing that mRNA vaccines can prevent infectious diseases in the future (Greaney et al., 2021). Various studies have shown that LNP technology can significantly boost the delivery of mRNA, thus providing intrinsic adjuvant activity and enhancing antigen expression.

Many antigens have already been found to be potential vaccine candidates against *T. gondii* like TgSAGs, TgROPs, TgMICs, among others (Mamaghani et al., 2023). However, the efficacy of different vaccine candidates cannot be compared because they were administered *via* different routes, animal types, vaccination doses, and vaccine production processes, all of which affect immunization efficacy (Zhang et al., 2022). In addition, there is lack of a uniform and standardized protocol for analysis of *T. gondii* vaccine efficacy. Previous studies have found that SAG1 is the predominant antigen on the surface of *T. gondii*, with strong immunogenicity and immunoprotective effects, and is considered a promising vaccine candidate (Pagheh et al., 2020). Hence, we chose SAG1 as a reference and compared efficacy of the predicted secreted antigens with linear B-cell epitopes and T-cell epitopes of SAG1 using a bioinformatics approach.

The search for vaccine candidate molecules were focused on the predicted *T. gondii* secretory proteins because of the essential role of these proteins in host-pathogen interactions (Xu et al., 2014). Secretory proteins containing transmembrane domains and signal peptides at the genome-wide level were screened on the *T. gondii* database (ToxoDB, http://ToxoDB.org). The screened candidates were then further analyzed for T/B cell epitopes.

Acquired immunity relies on T and B cells, which provide immune protection by recognizing antigen epitopes capable of forming pathogen-specific memory (Sasai et al., 2018; Khan and Moretto, 2022). The decoding of the whole genome of *T. gondii* provides a basis for subsequent analysis of T/B cell epitopes. Bioinformatics methods have been used to predict the epitopes of numerous *T. gondii* vaccine molecules, such as SAG4 (Zhou and Wang, 2017) and ROP21 (Zhang Z. et al., 2018). Herein, bioinformatics data showed that TG290 had a higher epitope score for linear B cells than SAG1 and a lower predicted IC50 value than SAG1, theoretically showing that TG290 is a promising vaccine candidate.

Humoral immunity is essential in resisting *T. gondii* infection. Antibodies can provide protective immunity response by regulating parasite phagocytosis, resisting invasion, and activating antibody-mediated classical complement pathway (Pifer and Yarovinsky, 2011). In this study, the levels of TG290-specific total IgG were increased in the serum of mice vaccinated with TG290 mRNA-LNP. In addition, TG290 mRNA-LNP significantly increased the levels of TG290-specific IgG1 and IgG2a antibodies, suggesting that TG290 mRNA-LNP can induce a mixed immune response of Th1/Th2.

IFN-γ improves host resistance to *T. gondii* infection (Park and Hunter, 2020). IFN-γ can also inhibit the propagation of *T. gondii*, activate macrophages, up-regulate NK cells, and promote the secretion of specific antibodies against *T. gondii* IgG1 and IgG2a *via* B cells (Boehm et al., 1997). IL-12 promotes IFN-γ production and T cell proliferation (Sun et al., 2020; Yu et al., 2021), while IL-4 promotes IFN-γ production late in infection (Tian et al., 2021). IL-10 can promote inhibition of inflammation and CD4 + T cells-mediated immunopathology (Wang et al., 2017). In this study, the levels of cytokines IFN-γ, IL-12, IL-4, and IL-10 were increased in mice inoculated with TG290 mRNA-LNP, consistent with previous studies (detection of *T. gondii* GRA24-based DNA vaccine; Zheng et al., 2019).

T-cell-mediated immune responses significantly prevent *T. gondii* invasion into host cell. Activating CD4^+^T lymphocytes depends on co-stimulators and MHC-II molecules, while the activation of CD8^+^T cells relies on APC or CD4^+^T helper cells (Verdon et al., 2020). T lymphocytes proliferate and differentiate after activation. Notably, T lymphocyte proliferation is widely used to reflect immune status (Luo et al., 2017). In this study, mice inoculated with TG290 mRNA-LNP vaccine produced significant T lymphocyte proliferation. Additionally, the type of immune response depends on the differentiation of T lymphocytes (Zhu et al., 2010). Cytotoxic T lymphocytes (CTLs) are differentiated from activated CD8^+^T cells by producing IFN-γ or perforin-mediated cytolysis (Halle et al., 2017). Activated CD4^+^T lymphocytes promote the activation of macrophages, recruitment of macrophages, and release of cytokines (Tubo and Jenkins, 2014). A previous study showed that vaccination with pVAX-MIC16 or pVAX-MIC5 induces T lymphocyte proliferation. In this study, immunization with pVAX-MIC16, pVAX-MIC5 or pVAX-MIC16 + pVAX-MIC15 significantly increased the percentage of CD4^+^ and CD8^+^ T cells compared with the control group (Zhu et al., 2021). Furthermore, the proportion of CD4^+^ and CD8^+^T lymphocytes significantly increased in mice vaccinated with the TG290 mRNA-LNP vaccine, consistent with previous studies.

DCs is a vital antigen-presenting cell crucial in activating innate and acquired immunity (Schraml, 2015). As an essential checkpoint of surface expression of mature DC, CD83 plays a pivotal role in regulating immunity and inducing inflammation regression (Grosche et al., 2020). Studies have also shown that CD83 significantly influence T cell stimulation (Pinho et al., 2014). CD86 is a key co-stimulatory molecule that binds to the CD28 molecule on the surface of T cells and provides a co-stimulatory signal to T cells, thus lowering the activated threshold of the initial T cells (Greenwald et al., 2005). Furthermore, CD86 molecules can regulate antigen presentation (Baravalle et al., 2011). Mature DC can also produce MHC-II molecules and mainly activate CD4^+^T cells (Fooksman, 2014). Meanwhile, MHC-I molecules are expressed in all nucleated cells and are crucial in antigen presentation at the endogenous level, thus promoting CD8^+^T cell activation (Lyons et al., 2001). MHC-I molecules can also deliver exogenous antigenic peptides to the cell surface *via* the cross-presentation pathway, thereby activating CD8^+^T cells (Colbert et al., 2020). In addition, the upregulation of MHC-I molecules may enhance IFN-γ secretion (Zhou, 2009). TgP2-pVAX1/PLGA and TgP2-pVAX1/CS increase the levels of CD83, CD86, MHC-II, and MHC-I molecules (Yu et al., 2022). In this study, the levels of CD83 and CD86 molecules were significantly increased in TG290 mRNA-LNP immunized mice, indicating that TG290 mRNA-LNP can promote DC maturity and enhance the expression of some co-stimulatory molecules. Moreover, the levels of MHC-II and MHC-I molecules were significantly elevated in mice immunized with TG290 mRNA-LNP. These results show that TG290 mRNA-LNP can facilitate DC maturation and stimulate MHC-I and MHC-II-dependent antigen presentation.

IRF8 is a crucial transcription factor that can modulate the expression of IL-12p40 and IL-12p35 in response to TLR11 and MYD88 activation (Tailor et al., 2006). In this study, the levels of IRF8 were significantly increased in mice immunized with TG290 mRNA-LNP, suggesting that TG290 mRNA-LNP vaccination may elicit IL-12 expression *via* IRF8 pathway. NF-κB is analogous to the IRF8 signaling pathway and is crucial in the generation of IL-12 or IFN-γ (Sangaré et al., 2019). In this study, the levels of NF-κB in Splenocytes were significantly increased in TG290 mRNA-LNP-immunized mice, indicating that TG290 mRNA-LNP immunity can induce the expression of IL-12 or IFN-γ through NF-kB signal pathway. T-bet regulates Th0-specific differentiation, which promotes Th1/Th2 exchange. Meanwhile, Th1 cells are selectively expressed by T-bet. In this study, TG290 mRNA-LNP immunologically evoked T-bet expression. These findings demonstrate that activation of the IRF8 and NF-κB pathways and T-Bet-mediated activation of CD4 + T cells and NK cells may elevate IFN-γ, similar to previous studies (Zhang N. Z. et al., 2018).

The percentage survival of vaccinated mice under a lethal challenge with *T. gondii* RH tachyzoites can be used to evaluate potential vaccine candidates. In this study, the mice vaccinated with TG290 mRNA-LNP had significantly extended survival time, while mice in the control group died within 8 days post challenge with type I RH tachyzoites (Figure 8A, 18.5 ± 3 days, *p* < 0.0001). Nevertheless, all mice eventually died. Similarly, a previous study also showed that TgCDPK1 can significantly prolong the survival time of mice (20% of these survived for 17 days) compared with the control groups (died within 8 days; Huang et al., 2019). Moreover, pVAX-MIC6 (11.5 ± 0.8 days), pVAX-GRA24 (8.1 ± 0.5 days), pVAX-GRA25 (9.4 ± 0.7 days), pVAX-GRA24 + pVAX-GRA25 (13.8 ± 0.9 days) and pVAX-GRA24 + PVAX-GRA25 + pVAX-MIC6 (18.7 ± 1.3 days) can significantly prolong the survival time of mice under lethal challenge (Xu et al., 2019). Notably, combination of vaccines may enhance the protective efficacy against *T. gondii*.

Furthermore, the survival time of mice adoptively immunized with serum (Figure 8B, 11.9 ± 2.6 days, *p* < 0.0001) and splenocytes (Figure 8C, 14.4 ± 2.5 days, *p* < 0.0001) was significantly prolonged compared with mice in the control groups (died within 8 days). Similarly, a previous study showed that vaccine-evoked immune serum and splenocytes can enhance resistance to *T. gondii* infection (Wang et al., 2020).

Bioinformatics analysis indicated that most regions of TG290 protein were flexible. Hydrophilicity plots showed that TG290 protein had an ideal antigenic index and surface probability, indicating that TG290 is a promising vaccine candidate. Further results also indicated that TG290 mRNA-LNP could elicit humoral and cellular immune responses, enhance cytokine production, evoke DCs and T lymphocytes, thus prolonging the survival time of mice vaccinated with TG290 mRNA-LNP. In summary, TG290 is a potential candidate molecule for the production of anti-*T. gondii* vaccine, providing a basis for the further design of TG290-based multi-epitope vaccines.

## Data availability statement

The datasets presented in this study can be found in online repositories. The names of the repository/repositories and accession number(s) can be found in the article/Supplementary material.

## Ethics statement

This study was approved by Hangzhou Medical College Institutional Animal Care and Use Committee (No: 2021-152) and followed the Chinese legislation regarding the use and care of research animals (GB/T35823-2018).

## Author contributions

BZ oversaw and conceptualized the project, designed the study, and edited and reviewed manuscript. DL, YZ, and SL performed experiments and raw data pre-processing. DL and YZ performed data analysis and drafted the manuscript. SL performed literature search. All authors contributed to the article and approved the submitted version.

## Funding

This work is supported by Zhejiang Medical and Health Science and Technology Plan (2020KY102), Scientific Research Project of Zhejiang Provincial Department of Education (Y202146047), Special Funding Program in Hangzhou Medical College (YS2021003), The Central Leading Local Science and Technology Development Fund Project For novel vaccine key technology research and platform construction.

## Conflict of interest

The authors declare that the research was conducted in the absence of any commercial or financial relationships that could be construed as a potential conflict of interest.

## Publisher’s note

All claims expressed in this article are solely those of the authors and do not necessarily represent those of their affiliated organizations, or those of the publisher, the editors and the reviewers. Any product that may be evaluated in this article, or claim that may be made by its manufacturer, is not guaranteed or endorsed by the publisher.

## Footnotes

^1^ https://swissmodel.expasy.org/

^2^ https://www.rcsb.org/

^3^ https://cluspro.bu.edu/
