# Supplementary material for: A novel Toxoplasma gondii TGGT1_316290 mRNA-LNP vaccine elicits protective immune response against toxoplasmosis in mice
Source: Front Microbiol. 2023 Mar 21;14:1145114. doi: 10.3389/fmicb.2023.1145114 (PMC10070739; doi:10.3389/fmicb.2023.1145114)

**BepiPred-2.0: Sequential B-Cell Epitope Predictor**

- Reference: [Jespersen MC, Peters B, Nielsen M, Marcatili P. BepiPred-2.0: improving sequence-based B-cell epitope prediction using conformational epitopes. Nucleic Acids Res 2017](https://www.ncbi.nlm.nih.gov/pubmed/28472356).
- The BepiPred-2.0 server predicts B-cell epitopes from a protein sequence, using a Random Forest algorithm trained on epitopes and non-epitope amino acids determined from crystal structures. A sequential prediction smoothing is performed afterwards. The residues with scores above the threshold (default value is 0.5) are predicted to be part of an epitope and colored in yellow on the graph (where Y-axes depicts residue scores and X-axes residue positions in the sequence).

**TGGT1_316290**

**Input Sequences**

| 1 | AETGRTDYVP PVPLEEAKPV TNAADGAGCC AVASDASKTQ AEAEKELAKT LKNLVETRDS |
| --- | --- |
| 61 | ANTVRIKNAI AKLVTQQQAE RLVSKAVEAK AKKDAEVAEA EVQALEKELA ALCEAHTKSE |
| 121 | QKAAQLDEQL LKTKEVAIEK AHKLSKSVEA ELDFVKREVQ QIQSALPKFR TQDAEAERKV |
| 181 | HQATADIQLD GVAAAVKEVE APSQVETAQL QTERAVAEEQ TAETDTQGSG KSSFISVHKR |
| 241 | KHRKHAGKLT P |

**Average:** 0.549   **Minimum:** 0.289   **Maximum:** 0.682


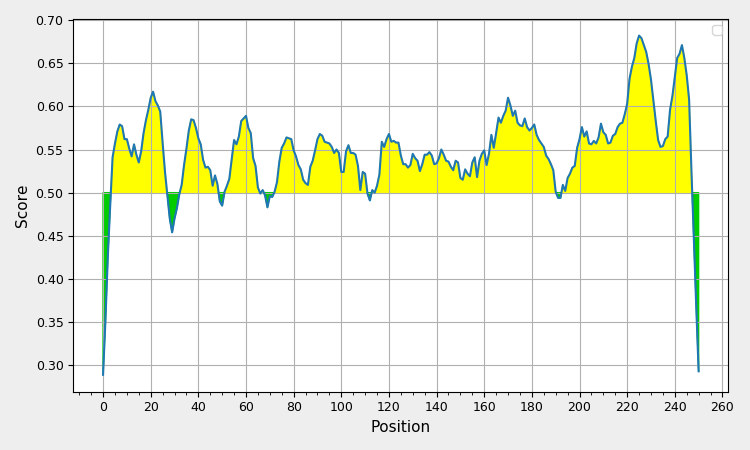


**Predicted peptides:**

| **No.** | **Start** | **End** | **Peptide** | **Length** |
| --- | --- | --- | --- | --- |
| 1 | 5 | 27 | RTDYVPPVPLEEAKPVTNAADGA | 23 |
| 2 | 34 | 49 | SDASKTQAEAEKELAK | 16 |
| 3 | 52 | 66 | KNLVETRDSANTVRI | 15 |
| 4 | 68 | 68 | N | 1 |
| 5 | 73 | 111 | LVTQQQAERLVSKAVEAKAKKDAEVAEAEVQALEKELAA | 39 |
| 6 | 114 | 191 | EAHTKSEQKAAQLDEQLLKTKEVAIEKAHKLSKSVEAELDFVKREVQQIQSALPKFRTQDAEAERKVHQATADIQLDG | 78 |
| 7 | 194 | 248 | AAVKEVEAPSQVETAQLQTERAVAEEQTAETDTQGSGKSSFISVHKRKHRKHAGK | 55 |

**SAG1**

**Input Sequences**

| 1 | MSVSLHHFII SSGFLTSMFP KAVRRAVTAG VFAAPTLMSF LRCGVMASDP PLVANQVVTC |
| --- | --- |
| 61 | PDKKSTAAVI LTPTENHFTL KCPKTALTEP PTLAYSPNRQ ICPAGTTSSC TSKAVTLSSL |
| 121 | IPEAEDSWWT GDSASLDTAG IKLTVPIEKF PVTTQTFVVG CIKGDDAQSC MVTVTVQARA |
| 181 | SSVVNNVARC SYGADSTLGP VKLSAEGPTT MTLVCGKDGV KVPQDNNQYC SGTTLTGCNE |
| 241 | KSFKDILPKL TENPWQGNAS SDKGATLTIK KEAFPAESKS VIIGCTGGSP EKHHCTVKLE |
| 301 | FAGAAGSAKS AAGTASHVSI FAMVIGLIGS IAACVA |

**Average: 0.479   Minimum: 0.226   Maximum: 0.631**


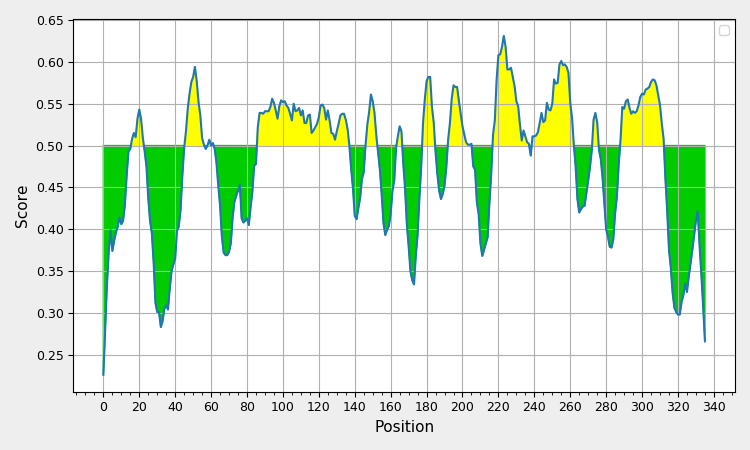


**Predicted peptides:**

| **No.** | **Start** | **End** | **Peptide** | **Length** |
| --- | --- | --- | --- | --- |
| 1 | 17 | 23 | SMFPKAV | 7 |
| 2 | 47 | 57 | ASDPPLVANQV | 11 |
| 3 | 59 | 62 | TCPD | 4 |
| 4 | 87 | 137 | LTEPPTLAYSPNRQICPAGTTSSCTSKAVTLSSLIPEAEDSWWTGDSASLD | 51 |
| 5 | 148 | 153 | EKFPVT | 6 |
| 6 | 165 | 167 | DDA | 3 |
| 7 | 179 | 185 | RASSVVN | 7 |
| 8 | 193 | 206 | GADSTLGPVKLSAE | 14 |
| 9 | 218 | 238 | DGVKVPQDNNQYCSGTTLTGC | 21 |
| 10 | 240 | 263 | EKSFKDILPKLTENPWQGNASSDK | 24 |
| 11 | 274 | 276 | FPA | 3 |
| 12 | 289 | 313 | SPEKHHCTVKLEFAGAAGSAKSAAG | 25 |

## Proteasomal Cleavage Prediction

NetChop is a predictor of proteasomal processing based upon a neural network. NetCTL is a predictor of T cell epitopes along a protein sequence. It also employs a neural network architecture. NetCTLpan is an update to the original NetCTL server that allows for prediction of CTL epitope with restriction to any MHC molecules of known protein sequence. The positive predictions are displayed in green, while the predictions below the threshold value are in red.

**TGGT1_316290**


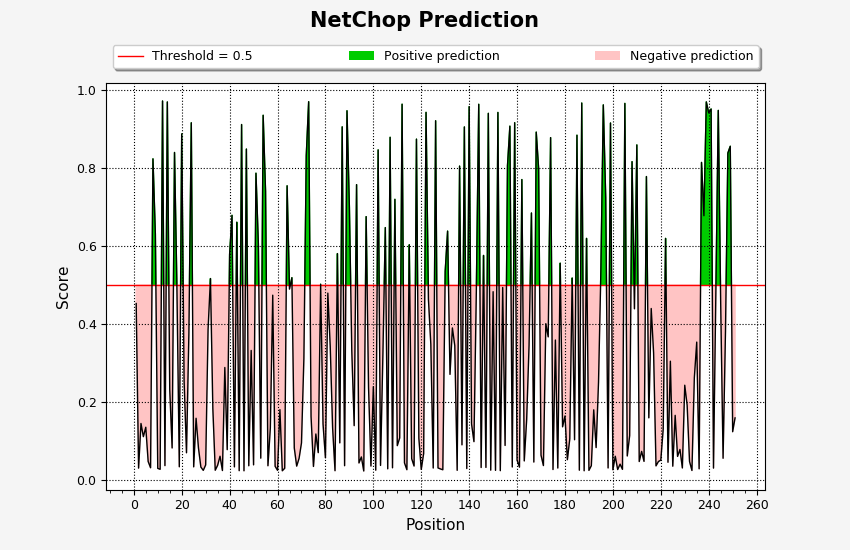


**SAG1**


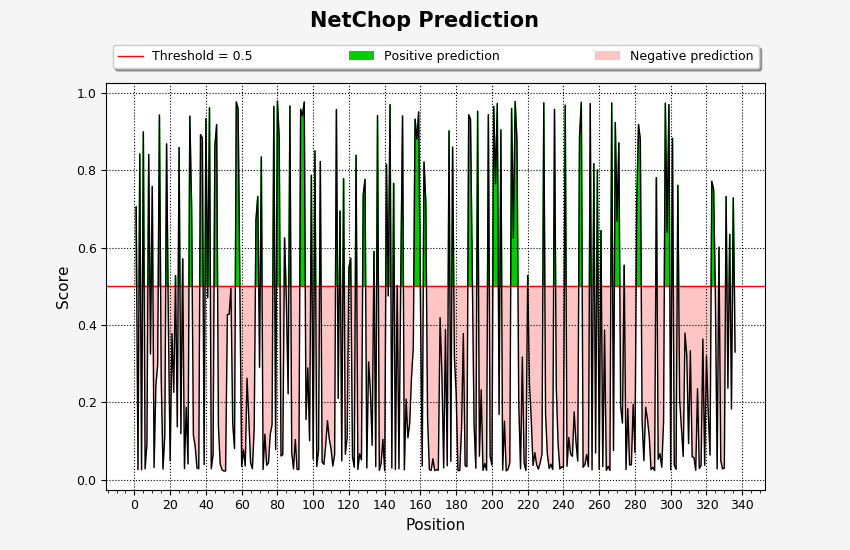

Supplement: Supplementary file 1 [file Data_Sheet_1.ZIP › supplementary materials/Results of BepiPred-2.0 and NetChop analyses.docx]
