# Supplementary material for: A novel Toxoplasma gondii TGGT1_316290 mRNA-LNP vaccine elicits protective immune response against toxoplasmosis in mice
Source: Front Microbiol. 2023 Mar 21;14:1145114. doi: 10.3389/fmicb.2023.1145114 (PMC10070739; doi:10.3389/fmicb.2023.1145114)
Supplement: Supplementary file 1 [file Data_Sheet_1.ZIP › supplementary materials/Results of Cluspro 2.0 protein-protein Docking.docx]

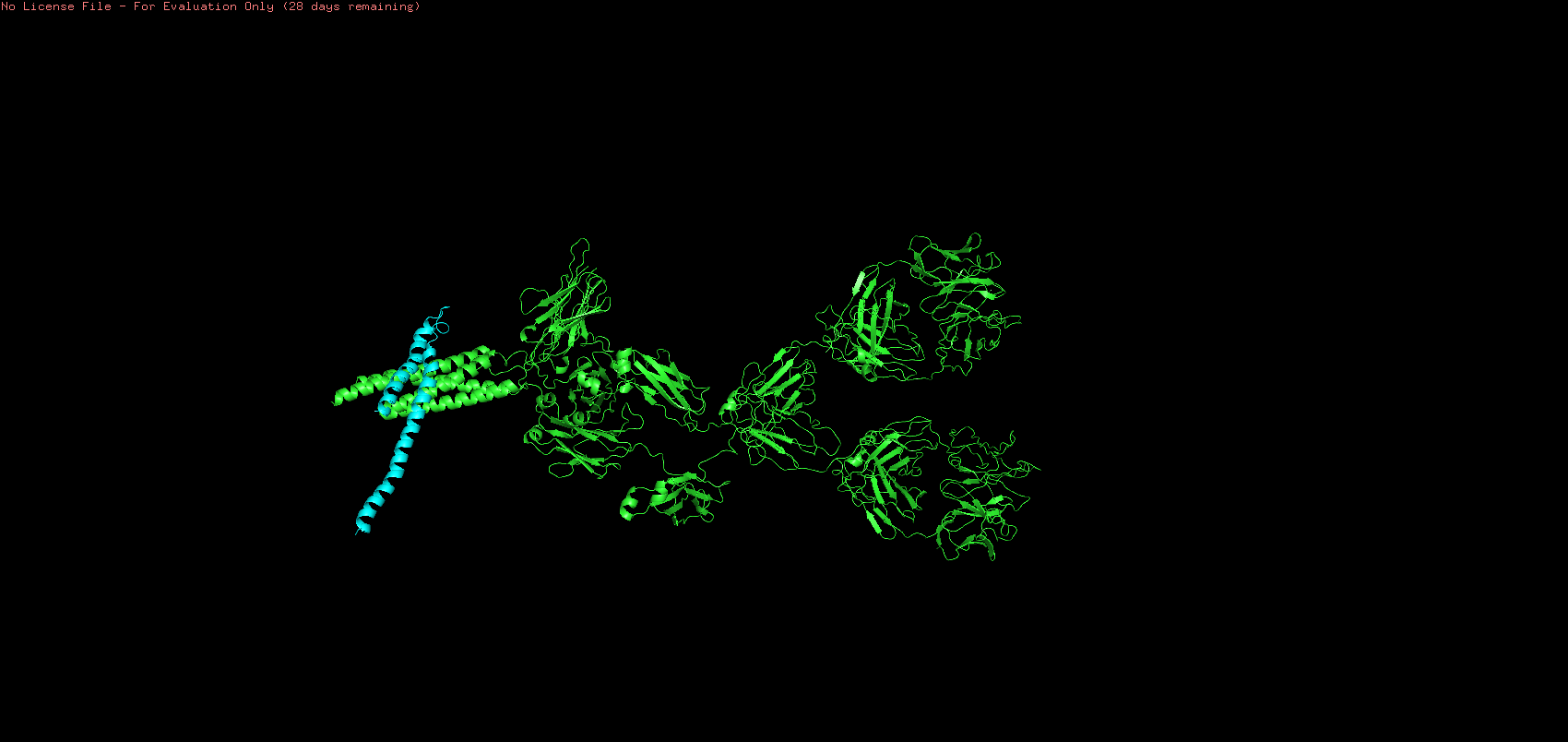


Fig.1 TG290-BCR protein-protein Docking


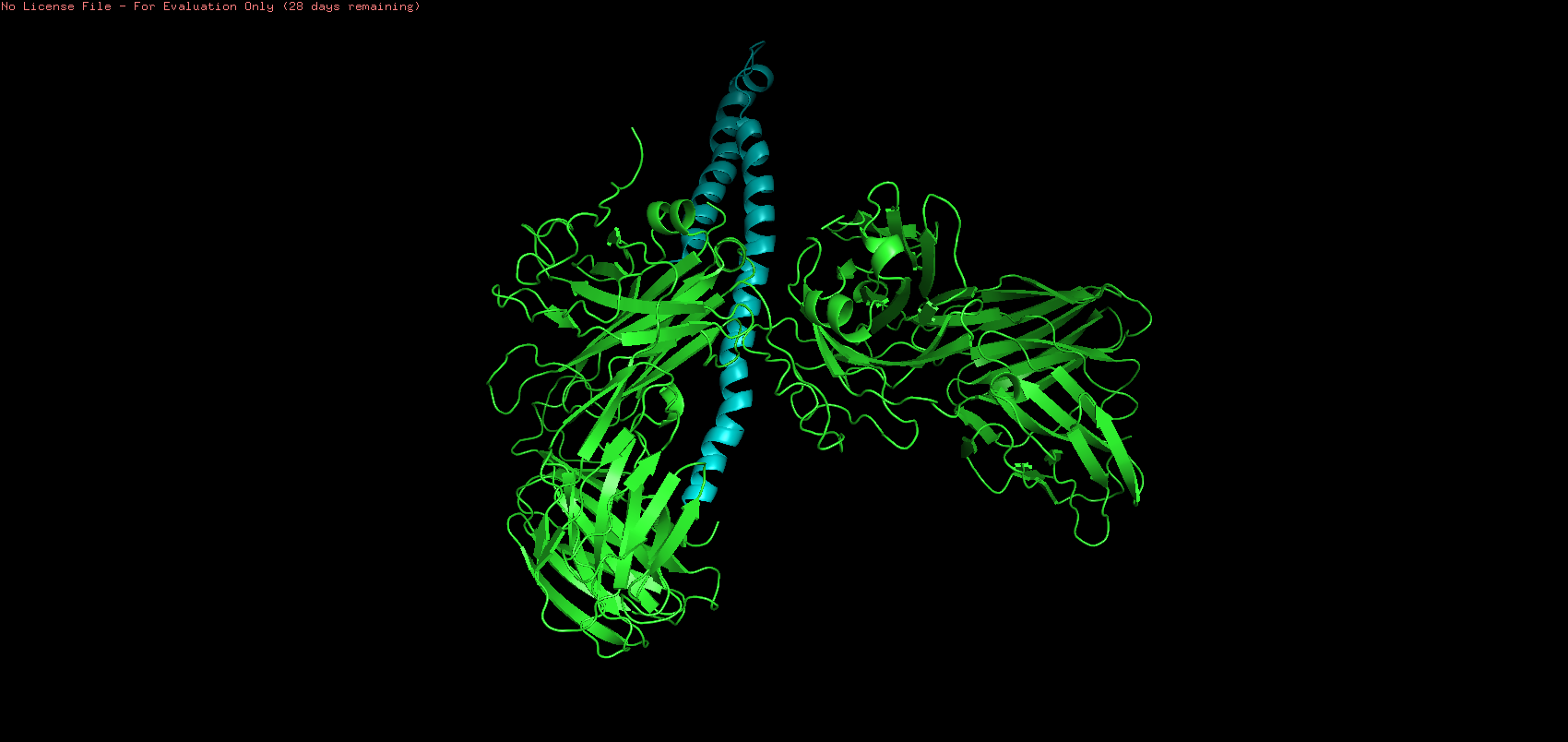


Fig.2 TG290-TCR protein-protein Docking


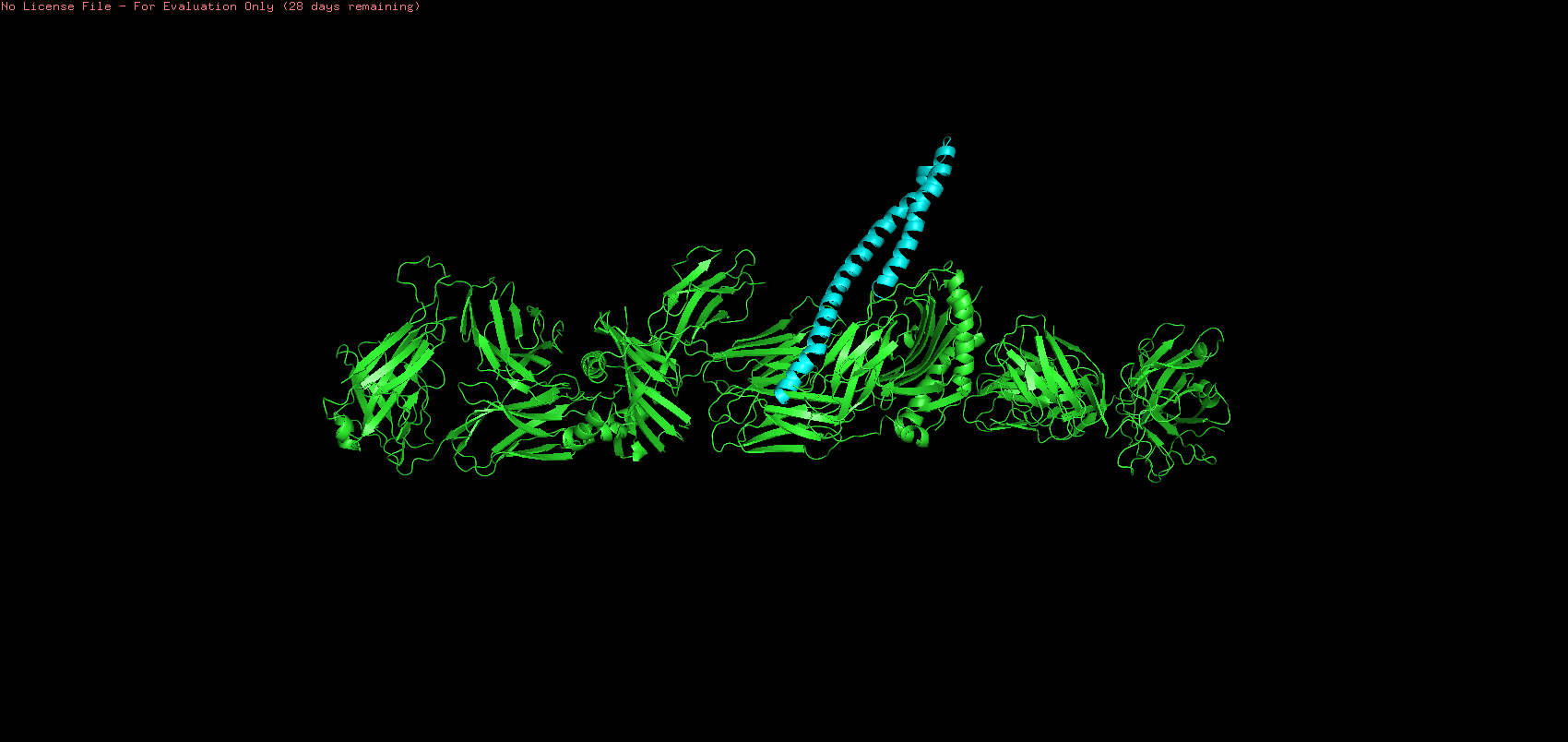


Fig.3 TG290-TCR-MHCII complex protein-protein Docking
