# Supplementary material for: A novel Toxoplasma gondii TGGT1_316290 mRNA-LNP vaccine elicits protective immune response against toxoplasmosis in mice
Source: Front Microbiol. 2023 Mar 21;14:1145114. doi: 10.3389/fmicb.2023.1145114 (PMC10070739; doi:10.3389/fmicb.2023.1145114)
Supplement: Supplementary file 1 [file Data_Sheet_1.ZIP › supplementary materials/Results of phylogenetic analyses.docx]

The protein sequence of TG_290 (accession number: TGGT1_316290) was obtained in ToxoDB (<http://toxodb.org/toxo/> ). We then performed a blastp (protein-protein BLAST) search on NCBI (<https://blast.ncbi.nlm.nih.gov/Blast.cgi> ) and obtained five sequence fragments (Figure 1). The MEGA software was used to construct a phylogenetic tree. After sequence alignment analysis, the sequences that could not be fully aligned at both ends were removed. The Neighbor-Joining Algorithm was used to construct the phylogenetic tree (Figure 2). The length of the tree branches can accurately represent genetic distance, and the order from closest to furthest is TGME49_316290, TGMAS_316290, TGCOUG_316290, TGRUB_316290, BN1204_058795.


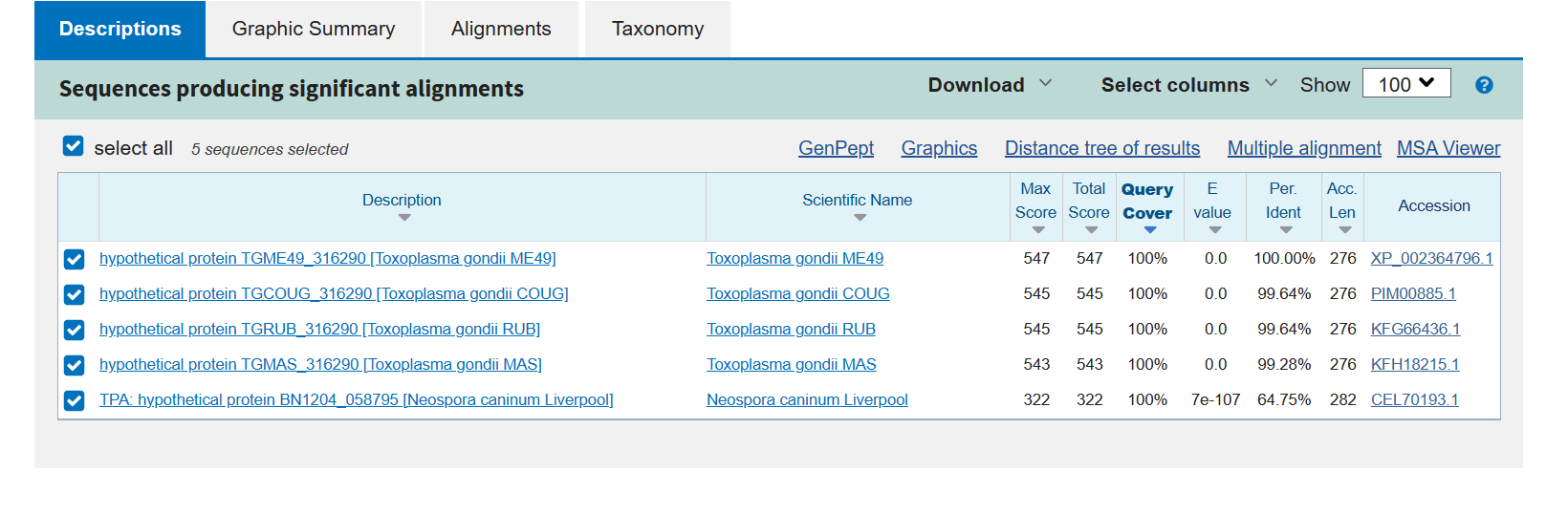


Figure 1 The sequence information obtained from NCBI

Figure 2 The phylogenetic tree of TGGT1_316290, TGME49_316290, TGMAS_316290, TGCOUG_316290, TGRUB_316290, BN1204_058795
